# **Electrical transport properties and Kondo effect in La**_**1−***x*_**Pr**_***x***_**NiO**_**3−**δ_**thin films**

**DOI:** 10.1038/s41598-021-84736-2

**Published:** 2021-03-08

**Authors:** Van Hien-Hoang, Nak-Kwan Chung, Heon-Jung Kim

**Affiliations:** 1 Department of Physics, Graduate School, Daegu University, Gyeongsan, Gyeongbuk 38453 Republic of Korea; 2 Research Team of Material Compatibility To Hydrogen Facility, Korea Research Institute of Standards and Science (KRISS), Daejeon, 34113 Republic of Korea; 3 Department of Materials-Energy Science and Engineering, College of Engineering, Daegu University, Gyeongsan, Gyeongbuk 38453 Republic of Korea

**Keywords:** Materials science, Condensed-matter physics, Nanoscale materials

## Abstract

The Kondo effect has been a topic of intense study because of its significant contribution to the development of theories and understanding of strongly correlated electron systems. In this work, we show that the Kondo effect is at work in La_1−*x*_Pr_*x*_NiO_3−δ_ (0 ≤ *x* ≤ 0.6) thin films. At low temperatures, the local magnetic moments of the 3*d e*_g_ electrons in Ni^2+^, which form because of oxygen vacancies, interact strongly with itinerant electrons, giving rise to an upturn in resistivity with *x* ≥ 0.2. Observation of negative magnetoresistance, described by the Khosla and Fisher model, further supports the Kondo picture. This case represents a rare example of the Kondo effect, where Ni^2+^ acts as an impurity in the background of Ni^3+^. We suggest that when Ni^2+^ does not participate in the regular lattice, it provides the local magnetic moments needed to scatter the conduction electrons in the Kondo effect. These results offer insights into emergent transport behaviors in metallic nickelates with mixed Ni^3+^ and Ni^2+^ ions, as well as structural disorder.

## Introduction

The Kondo effect was proposed by Jun Kondo in 1964^1^ and describes the resonant interaction between conduction charge carriers and the spin of localized magnetic impurity ions, manifesting as an anomalous upturn in resistivity at low temperatures. Although the Kondo effect was first discovered in conventional metals containing small concentrations of magnetic impurities, recent studies suggest its existence in several oxides and carbon-based materials such as SrTiO_3_^2^, graphene^3^, doped TiO_2_ thin films^4^, etc. These observations have inspired important further development and refinements of the Kondo physics.

The nickelate *R*NiO_3_ (*R* = rare earth element) is well known for its metal–insulator transition (MIT) and the accompanying structural phase transition that occurs when the temperature or the radius of the rare-earth ion is varied. Among them, LaNiO_3_ (LNO), which possesses a rhombohedral structure with a R-3c space group and has a pseudocubic lattice parameter of *a* = 3.838 Å with small lattice distortion is quite interesting, because LNO does not undergo the MIT in its bulk form. Indeed, it displays a metallic behavior over the entire temperature range^5,6^. However, when its thickness is reduced below 7 unit cells (u.c.) to an ultrathin LNO, a temperature-driven MIT has been observed^7^. In contrast, another *R*NiO_3_ (*R* = Pr, Nd, Sm, Eu) is orthorhombic with a $Pbnm$ space group in the high-temperature metallic phase, and its structure becomes monoclinic with $P2_{1} {/}n$ in the low-temperature insulating phase^8^. This suggests a structural distortion must accompany the temperature-driven MIT of the ultrathin LNO.

Bulk^5^ and thin film LNO’s^7,9–12^ have been extensively studied, and exhibit various correlation effects which originate with the large Hubbard U and relatively narrow conduction bandwidth^13,14^. In LNO, the nickel ions are considered to be in the Ni^3+^ valence state with a *d*^7^ configuration, where *d* denotes the *d* orbitals. The cubic crystal field effect lifts the degeneracy of the *d* orbitals into triply degenerate lower *t*_2g_ and doubly degenerate higher *e*_g_ levels, leading to a $t_{2g}^{6} e_{g}^{1}$ configuration in this ionic picture. This picture, however, has been challenged by recent works, which have suggested that Ni has a valence closer to 2+ with holes in the oxygen 2*p* band due to negative charge transfer energy. Thus, the ground state of the paramagnetic state is more adequately described as a $d^{8} \underline{L}$ configuration (where $\underline{L}$ denotes a ligand hole)^15^.

When an extra electron is added to LNO, for instance, by the formation of an oxygen vacancy, it annihilates the ligand hole, leaving two electrons in the *e*_g_ level, giving rise to the S = 1 impurity state. Since the presence and absence of the ligand hole results in shrinkage and expansion of the NiO_6_ octahedron, respectively, it is likely that the extra electrons occupy a defect site in the enlarged NiO_6_ octahedron. This defect carries a magnetic moment with S = 1 and becomes an electron scattering center. Because of this, LNO is highly sensitive to oxygen vacancies with extra electrons.

An oxygen vacancy combined with structural disorder is known to result in various phenomena such as localizations, metal–insulator transition, antiferromagnetic ordering, and the spin glass behavior^16–21^. In particular, we believe that the Ni^2+^ ions not participating in the regular lattice play an important role as magnetic impurities. The electrons that are trapped in such an impurity are movable, but the time scale for this hopping is expected to be much smaller than that of itinerant electrons. Thus, the magnetic moments of Ni^2+^ ions are considered to be quasi-static with respect to itinerant electrons.

Until now, however, very few systematic studies of LNO substituted with other trivalent rare-earth ions have been conducted, particularly regarding the effects of oxygen vacancies and increased disorder. In this work, the structural characteristics and transport properties of La_1−*x*_Pr_*x*_NiO_3−δ_ (LPNO)/SrTiO_3_ (STO) (001) thin films (*x* = 0, 0.1, 0.2, 0.3, 0.4, 0.5, 0.6) grown using a pulsed laser deposition (PLD) method were systematically investigated. We succeeded in preparing LPNO films with oxygen vacancies that varied nonmonotonically with *x* and with a maximum around *x* ~ 0.2–0.3. This set of films enabled us to correlate electrical transport properties with oxygen vacancy content. Interestingly, a pronounced low-temperature resistivity upturn was observed in the films with *x* ~ 0.2–0.3. Magnetoresistance (*MR*) measurements also revealed the largest negative *MR* at *x* ~ 0.2–0.3. These results support the Kondo picture, demonstrating that LPNO is a Kondo system, in which Ni^2+^ not participating in the regular lattice becomes a magnetic impurity.

## Methods and materials

LPNO films with 60 nm thickness were deposited on STO (001) substrates (Crystec) using the PLD method. The LPNO polycrystalline targets were synthesized using a sol–gel method. Details of the target synthesis are reported elsewhere^6^. A KrF excimer laser with a wavelength of 248 nm was used to ablate a LPNO target, with a repetition rate of 2 Hz, an energy density of 1 J/cm^2^ in an oxygen partial pressure of 0.1 Torr and at the substrate temperature of 750 °C. After deposition, the samples were examined using X-ray diffraction (XRD), X-ray photoelectron spectroscopy (XPS), resistivity, and *MR* measurements, which allowed the characterization of their crystalline, electronic, and electrical transport properties. XRD measurements of the LPNO epitaxial thin films were carried out by using synchrotron radiation at the 3A beamline at the Pohang Light Source (PLS). The temperature dependence of the electrical resistivity was measured in the temperature range from 1.7 to 300 K using a cryogen-free magnetic system (Cryogenic Inc.). Resistivity as a function of magnetic field *B* was measured between *B* = − 9 T and *B* = 9 T. *B* was applied perpendicular to the surface of the films. The Van der Pauw method was used for the resistance and *MR* measurements.

## Results and discussion

**Figure 1:**
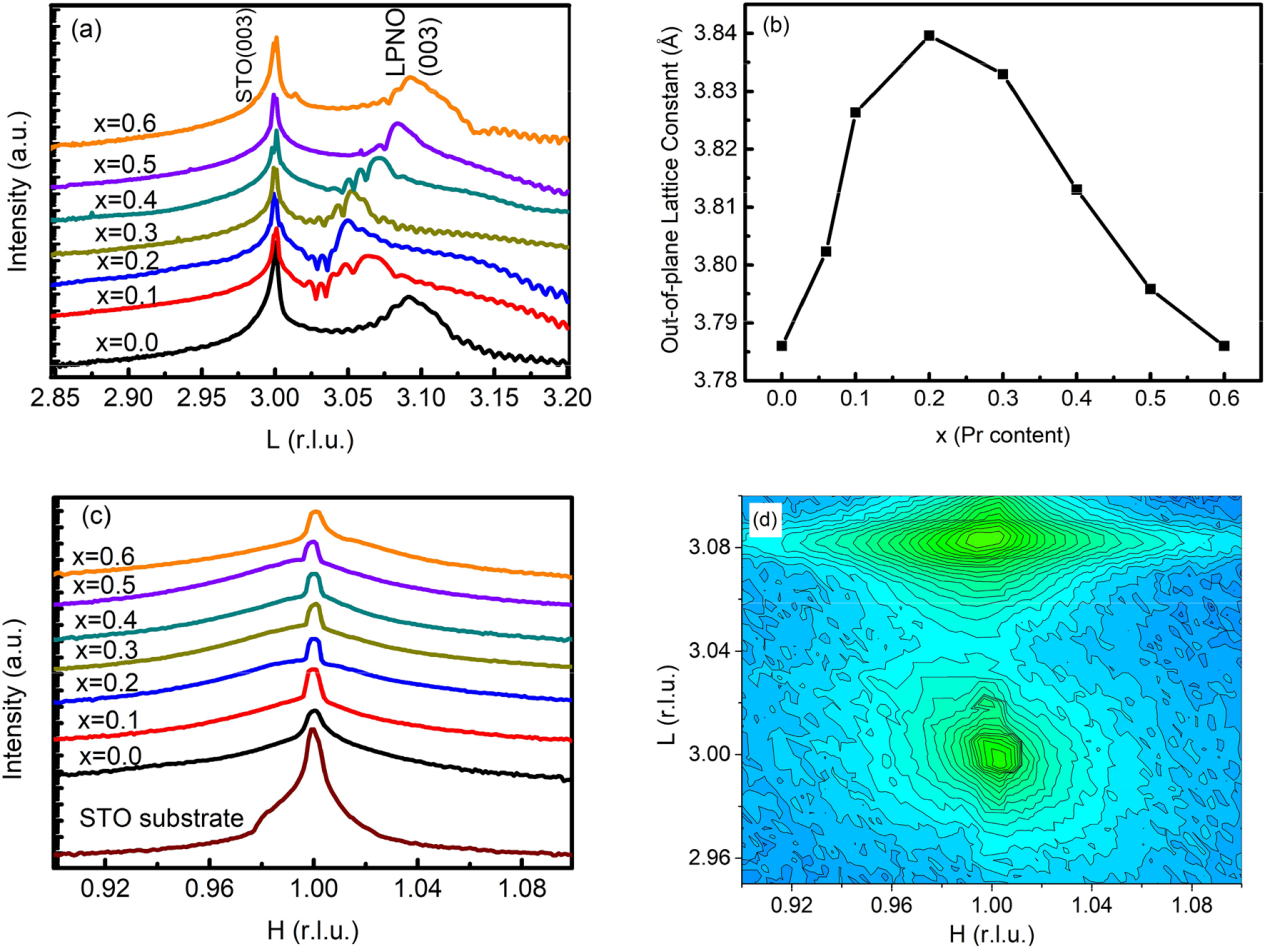
(**a**) X-ray scattering patterns for LPNO films scanned along the (00*l*) direction. (**b**) The *c* lattice constant as a function of the Pr concentration, estimated by X-ray scattering. (**c**) H scan from (0.9 0 L) to (1.1 0 L), where L is the L position of the film peak. (**d**) Reciprocal space mapping (RSM) around the (103) Bragg peak for the sample with *x* = 0.2.

Figure 1a shows the specular X-ray diffraction patterns of all the LPNO samples with different Pr concentrations *x*. All of the films were confirmed to be aligned along the [00*l*] directions. The films exhibited well-defined Kiessig fringes around the main Bragg peaks, indicating the smoothness of the interfaces and the surface at atomic scale. X-ray diffraction results also revealed a pure LPNO phase without any detectable secondary phase. All film peaks were located on the right side of the corresponding STO (00*l*) peak, meaning that the out-of-plane lattice parameter $c_{film}$ of the LPNO layer was smaller than that of the STO substrate (*a*_sub._ = 3.905 Å). From the peak positions, the $c_{film}$ lattice constant of the films was estimated, as summarized in Fig. 1b. Surprisingly, as shown in Fig. 1b, the *c* parameter underwent an increase below *x* = 0.2 and then a decrease above *x* = 0.2 with increasing *x*. Obviously, this is a violation of Vegard's law. According to Vegard's law, the *c* lattice constant should decrease with increasing *x* because the radius of the Pr^3+^ ion is smaller than that of the La^3+^ ion. However, that was not observed here.

One possible explanation for this deviation is that there was a different in-plane strain state for different *x*. In order to check the in-plane lattice constant *a* of the LPNO films, we performed H scans of all samples from (0.9 0 L) to (1.1 0 L), where L corresponds to the peak position in the L-scan of each sample, as shown in Fig. 1a. Figure 1c presents the results of the H scan. The peak positions of the H scan barely changed in all the samples, indicating full in-plane strain.

We also carried out reciprocal space mapping (RSM) for *x* = 0.2. Figure 1d presents the RSM for a sample with *x* = 0.2 around the (103) asymmetric reflection. As shown in Fig. 1d, the H value of the LPNO diffraction peak was the same as that of the STO substrate due to the full strain offered by the substrate. This result was quite consistent with the results of the H scans. Accordingly, the present results imply that the unit cell volume expands significantly, and unusually follows the same *x* dependence as the *c* lattice constant. We tentatively attribute this behavior to the different amounts of oxygen vacancies in the samples. This is possible because fully strained films with different Pr concentrations *x* can accommodate different amounts of oxygen vacancies.

In this case, the *x* dependence of the *c* lattice constant reflects oxygen vacancy concentrations. Because oxygen vacancies, which act as a donor, increases the cell volume, the sample with *x* = 0.2, which has the largest cell volume, should have the largest amount of oxygen vacancies as well as resultant Ni^2+^ ions. On the other hand, disorder introduced by substitution of La atoms by Pr atoms is of secondary importance because the difference in atomic radius between La and Pr is small. If this were the dominant effect, Vagard’s law should hold.

**Figure 2:**
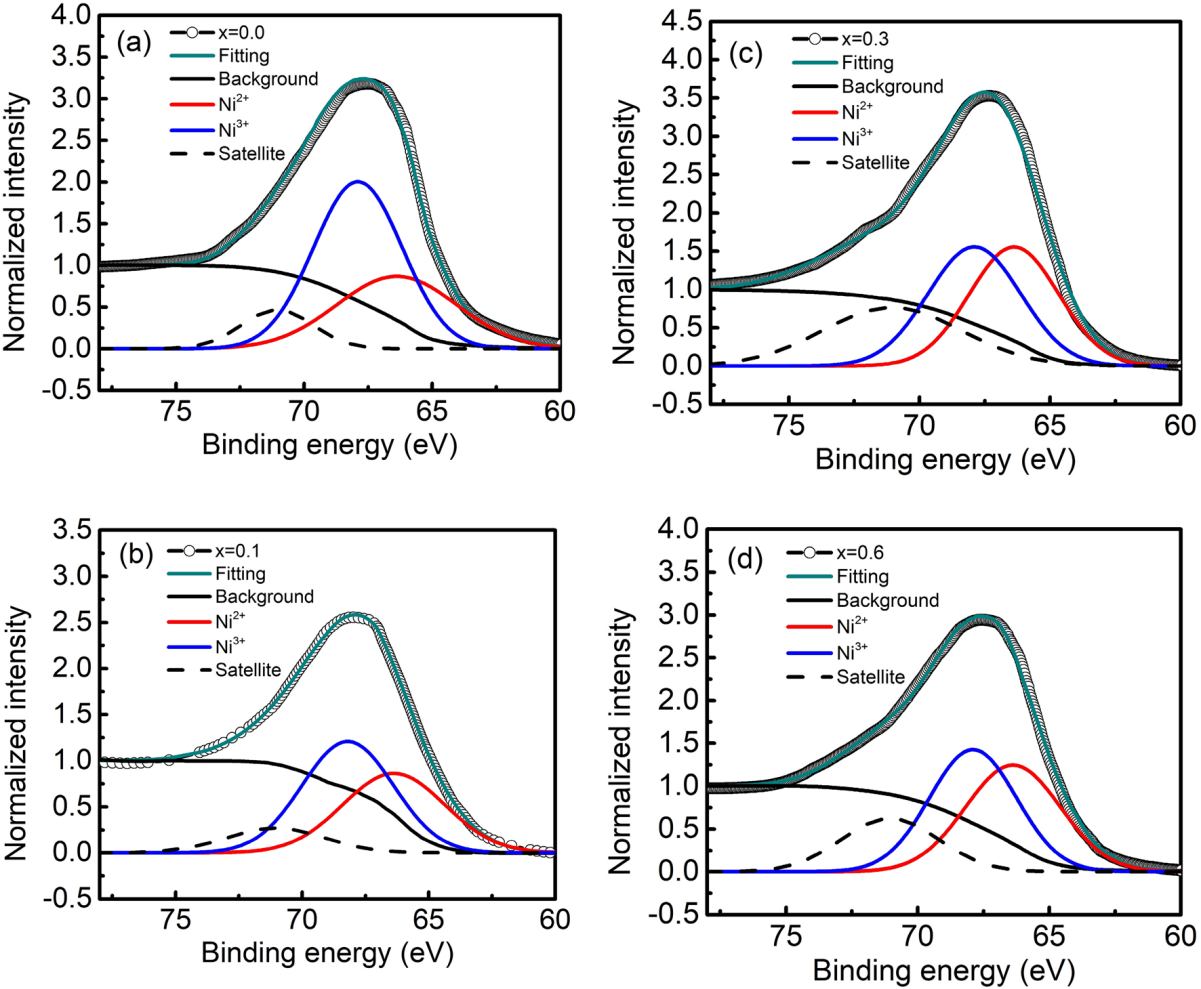
XPS spectra at the Ni 3*p* edge of the films with (**a**) *x* = 0.0, (**b**) *x* = 0.1, (**c**) *x* = 0.3, and (**d**) *x* = 0.6. The blue (red) solid and dashed lines represent the fitted curves of the Ni^3+^ (Ni^2+^) and satellite peaks, respectively.

In order to determine the Ni oxidation state of the films, XPS measurements were carried out for *x* = 0.0, 0.1, 0.3, and 0.6. All measured samples show overlap of several Ni 3*p* peaks within the range of 60–75 eV, which is in good agreement with the previous reports nominally associated with Ni^3+^ and Ni^2+^ 3*p* binding energies^22,23^. To ensure the quality of the peak fitting, a Shirley background and a combined Lorentzian-Gaussian function were used. The fitting results for samples are presented in Fig. 2 and Table 1. As shown in Fig. 2, the observed spectra are deconvoluted into three components i.e. Ni^2+^ (red), Ni^3+^ (blue), and satellite (black dash). The relative area under the red and blue fitting curves reflects the Ni^2+^/Ni^3+^ ratio in the films^20,24^. The obtained results (Table 1) clearly show that the Ni^2+^/Ni^3+^ ratio increases monotonically with the increase in *x* below *x* < 0.3. The maximum value is calculated to be 0.96 at *x* = 0.3, and then the ratio decreases above *x* = 0.3 with increasing *x*. The XPS result is in qualitative agreement with *x* dependence of the *c* lattice constant.

**Table 1:** The ratio of Ni^2+^ to Ni^3+^ of samples with *x* = 0.0, 0.1, 0.3, and 0.6 calculated from Ni 3*p* XPS spectra in Fig. 2.

Pr content *x*	Peak position (eV)		Relative area of Ni^2+^	Relative area of Ni^3+^	Ni^2+^/Ni^3+^ ratio
	Ni^2+^	Ni^3+^			
0.0	66.38	67.98	5.11	8.7	0.60
0.1	66.37	67.95	4.36	5.47	0.80
0.3	66.40	67.99	6.66	6.93	0.96
0.6	66.39	67.99	5.28	5.91	0.89

Next, we discuss the electrical transport properties of the LPNO films. To understand the electrical transport behaviors of the LPNO thin films, we measured resistivity as a function of temperature *T* as shown in Fig. 2a. The films with *x* = 0 and *x* = 0.1 were metallic at all temperatures. For LNO (*x* = 0), the resistivity behavior was similar to those previously reported^7,25^. In contrast, the samples with *x* ≥ 0.2 exhibited an upturn in resistivity at low temperatures.

Quite interestingly, we observed that the degree of the upturn varied nonmonotonically with the increase in *x* concentration; the films with intermediate Pr concentrations (*x* = 0.2 and *x* = 0.3) exhibited a large upturn, while the upturns in the samples with other Pr concentration were less pronounced. This observation can be attributed to the different amounts of disorder induced by the different electronegativities of La^3+^ and Pr^3+^, or the oxygen nonstoichiometry^6^. This upturn behavior was quite well correlated with the *x* dependence of the *c* lattice constant deduced from the X-ray results.

To further explore the effect of Pr concentration *x* on electrical transport behaviors, we first analyzed the metallic part of the resistivity. Nickelate is often considered to be a non-Fermi liquid (NFL)^26–28^, whose resistivity temperature dependence (*T*) is as follows,

1 $$\rho_{(NFL)} = \rho_{0} + AT^{n} ,$$

with an exponent *n* < 2^10,29,30^, where $\rho_{0}$ is the residual resistivity and $A$ is a coefficient that measures the strength of electron–electron scattering. For a Landau Fermi liquid (LFL), on the other hand, the exponent *n* equals 2. The relationship between elastic and inelastic scattering can be described in terms of the mean free path, $l$, where $l$ is given by^10^

2 $$\frac{1}{l} = \frac{1}{{l_{e} }} + \frac{1}{{l_{in} }},$$

where $l_{e}$ and $l_{in}$ are the elastic and inelastic components of the mean free path, respectively. $l_{in}$ decreases with increasing temperature, resulting in an increase in resistivity. However, the value of $l_{in}$ cannot be smaller than the lattice constant. The regime where the mean free path is comparable to lattice spacing is called the Ioffe–Regel limit.

Equation (1), however, only describes experimental data for a limited range of temperatures^31^. $\rho (T)$ is fully described when the resistivity saturation $\rho_{SAT}$ is taken into account^10,29,30,32,33^, and $\rho (T)$ is given by

3 $$\rho^{ - 1} (T) = \rho^{ - 1}_{NFL} + \rho^{ - 1}_{SAT} ,$$

Equation (3) is called the parallel resistor model and this phenomenological model has been applied to a wide range of materials including elemental metals, heavy fermion compounds, and alloys^34,35^. Here $\rho_{SAT}$ is interpreted as the resistivity at high temperatures where the Ioffe–Regel limit is reached^36^.

**Figure 3:**
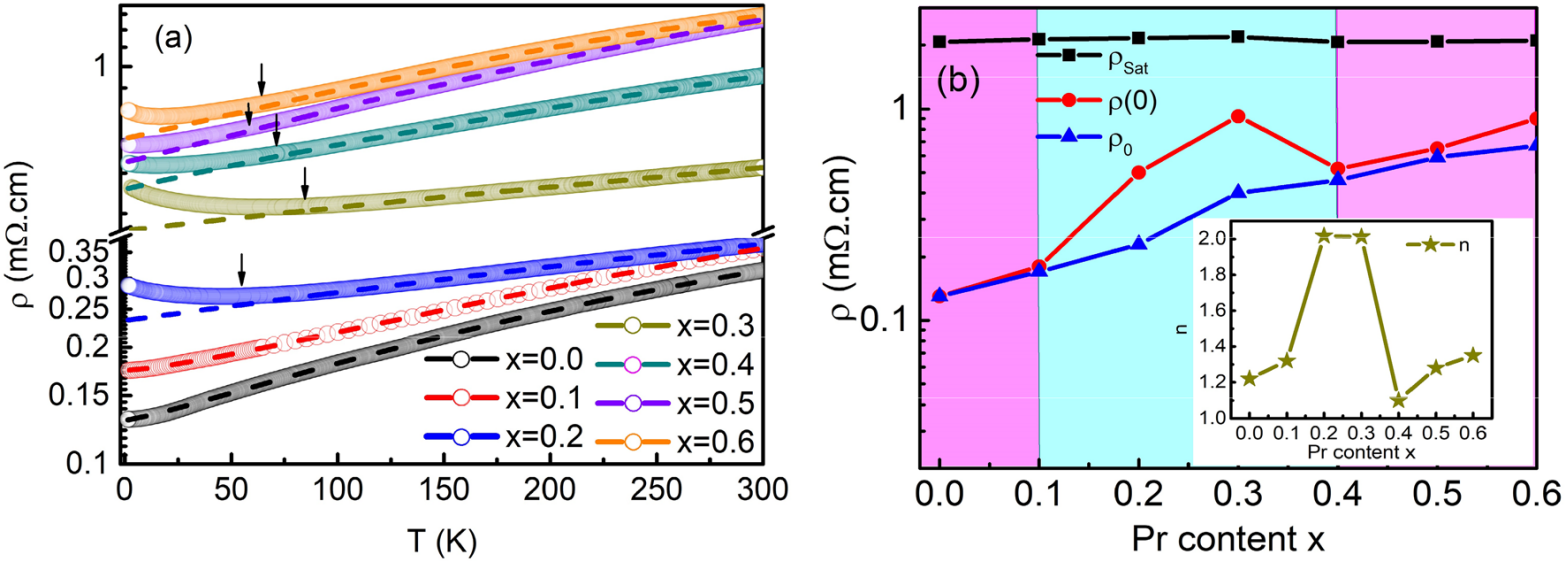
(**a**) The resistivity of epitaxial LPNO films grown on STO (001) substrates as a function of temperature. The dashed lines are fits of data in the metallic region using Eqs. (1) and (3). Arrows indicate the temperature at which the upturn starts. (**b**) $\rho_{0}$, $\rho_{SAT}$ and n (inset) are the fitting parameters extracted from fitting curves and $\rho \left( 0 \right)$ is the metallic resistivity extrapolated to 0 K. The region with the pink color indicates metallic behavior. The region with light blue represents a large upturn in resistivity.

A combination of Eqs. (1) and (3) can explain the *T* dependence of the resistivity in the metallic regime for all the LPNO films. The fitting results are shown as dashed lines in Fig. 3a, and the obtained fitting parameters are summarized in Fig. 3b. Here we define $\rho (0)$ as the resistivity extrapolated to *T* = 0 K. When the samples are metallic at all measured temperatures, $\rho (0)$ is nearly equal to the residual resistivity $\rho_{0}$. On the other hand, in the samples with a resistivity upturn, $\rho (0)$ should be larger than $\rho_{0}$. In a metal, it is well-known that $\rho_{0}$ reflects the density of defects and is correlated with the elastic mean free path. However, this does not hold when there is an upturn.

The difference between $\rho (0)$ and $\rho_{0}$ was quite pronounced for the films with *x* = 0.2 and *x* = 0.3 as shown in Fig. 3b. Interestingly, these two samples exhibited *n* ~ 2, the exponent of the LFL, while other samples had the exponent *n,* which is smaller than 2 (the inset of Fig. 3b). These observations suggest that the samples were all on the verge of a localization; the electrons were localized at low temperatures in *x* = 0.2 and *x* = 0.3, and they nearly were at other concentrations. Therefore, an exponent *n* smaller than 2 may be an indication of a disorder-induced NFL state, which is stabilized just before the localization induced by the Kondo effect occurs. In the disorder-induced NFL state, the disorder-induced distribution of the Kondo temperature down to T = 0 K results in unscreened local moments and NFL physics. The nature of the localization will be discussed later.

The saturation resistivity is relatively independent of the Pr concentration *x*. $\rho_{SAT}$ in nickelate films was reported to be sensitive only to the in-plane strain^29^ due to the modification of bands near the Fermi energy^37^. In our fully strained films, the in-plane lattice parameter was fixed at the value of the STO substrate. This may be the reason why $\rho_{SAT}$ did not vary with the Pr concentration *x*.

To demonstrate that all samples were indeed in the Ioffe–Regel limit at *T* = 300 K, we estimated the mean free path at *T* = 300 K, for instance at *x* = 0.3 using the formula^29,32^ of

4 $$l(T) = \frac{{3\pi^{2} \hbar }}{{q^{2} k_{F}^{2} \rho (T)}},$$

where $\hbar$ is the reduced Planck constant, $q$ is the elementary charge, $\rho (T)$ is the resistivity at *T*, and $k_{F}$ is the Fermi wave vector. In a spherical Fermi surface, $k_{F}$ is given by $(3\pi^{2} n)^{1/3}$, where $n$ is approximately the carrier density of LNO, and $n = 1.31 \times 10^{22} \;{\text{cm}}^{ - 3}$^10^. From Eq. (4), the mean free path at *T* = 300 K for *x* = 0.3 is estimated to be 3.990 Å. This value is close to the bulk LNO lattice constant of *a* = 3.838 Å. Therefore, this confirms that our films were indeed in the Ioffe–Regel limit at *T* = 300 K and justifies our assumption of resistivity saturation.

To understand the origin of the resistivity upturns observed at low temperatures, we analyzed our experimental data based on four different mechanisms; (1) Spin polarized tunneling through grain boundaries, (2) quantum interference effect (QIE) such as weak localization (WL), (3) localization caused by the electron–electron interaction (EEI), and (4) the Kondo effect based on spin dependent scattering.

Grain boundary tunneling plays a key role in granular samples at low temperatures^38^. Upon a decrease in temperature, the charge transfer of electrons with definite spins through grain boundaries is prohibited, due to spin freezing, and spins are confined in the individual grains. Consequently, below a certain temperature the resistivity rises. However, this phenomenon only occurs in polycrystalline and granular samples^38^. Therefore, we can rule out this mechanism.

On the other hand, weak localization arises from the quantum interference effect between the clockwise and counter-clockwise travelling wave of an electron in a closed path. The applied magnetic field breaks the interference condition, leading to the suppression of the resistivity minimum^39^. Since the weak localization is more pronounced in a two-dimensional system, it is of less importance in a thick film or a bulk sample, but in an ultra-thin film, it is a predominant effect^7,38^. In addition, in our case, the resistivity upturn did not appear for the pure LNO sample (*x* = 0). In previous reports, the resistivity upturn was observed in a LNO film, and its occurrence was attributed to weak localization^7^. Since nonmagnetic impurities can play an important role in weak localization, the absence of any upturn in our LNO sample suggests nonmagnetic impurities were not a significant influence. Moreover, because magnetic Ni^2+^ induced by oxygen vacancies is believed to be a dominant impurity, the weak localization picture is not relevant.

To further confirm the absence of the weak localization in our samples, we carried out fitting the low *T*-upturn part of resistivity for *x* = 0.2 and magnetorresistance curve for *x* = 0.3, respectively using Eqs. (5) and (6) below. In a three-dimensional (3D) system, the WL conductivity and magnetoresistance can be expressed as following^40,41^; for T dependence of resistivity,

5 $$\Delta \sigma (T) = \sigma_{0} + CT^{2}$$

Here $\sigma_{0}$ is residual conductivity and $C$ is the coefficient of the WL term.

**Figure 4:**
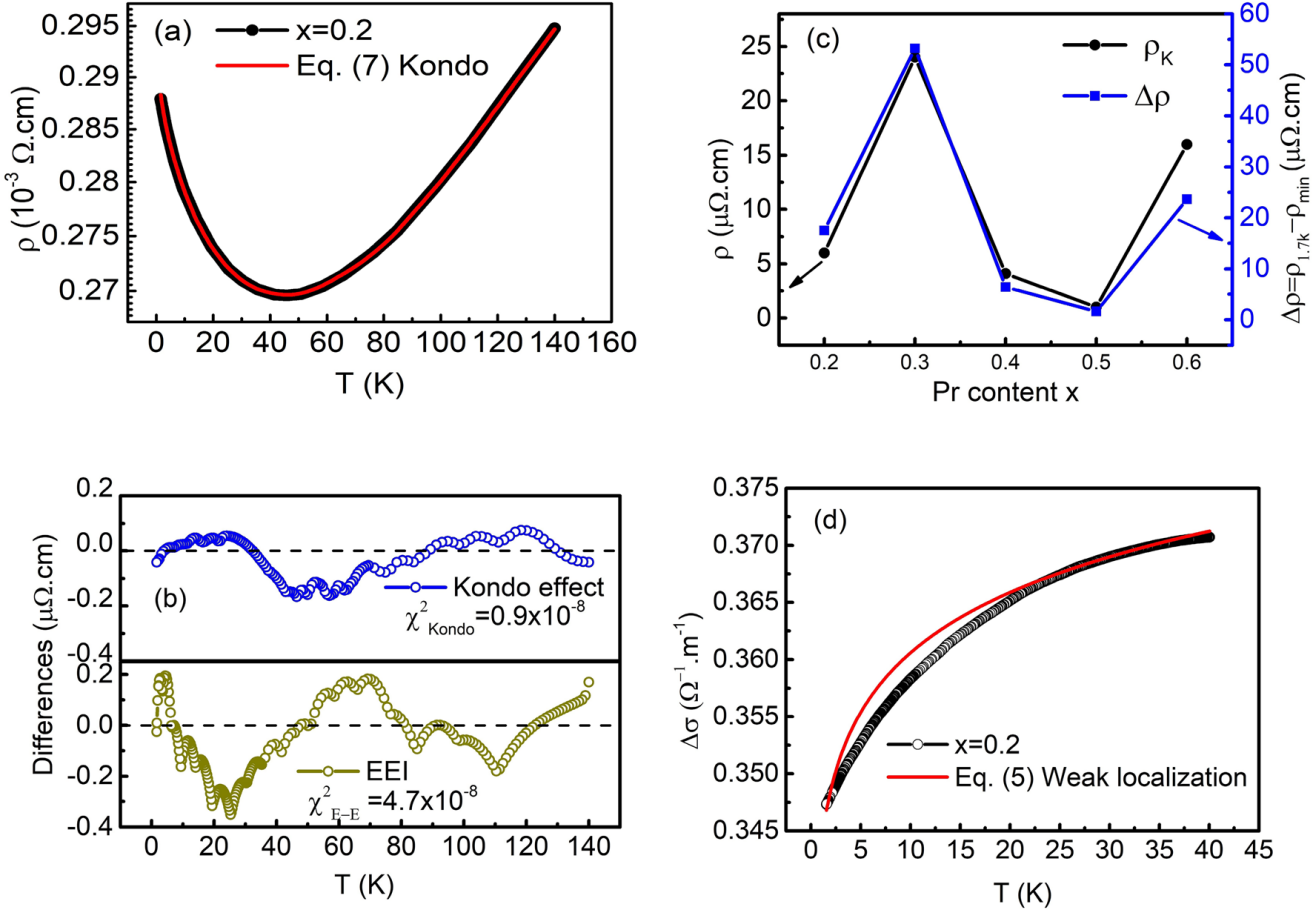
(**a**) Upturn component of the resistivity as a function of temperature for LPNO with *x* = 0.2. The dashed line is the curve fit using Eq. (7). (**b**) The difference in curves for *x* = 0.2 between the experimental data and theoretical fitting calculated based on Eqs. (7) and (8), respectively. (**c**) The Kondo coefficient $\rho_{k}$ and $\Delta \rho = \rho_{{1.7\;{\text{K}}}} - \rho_{\min }$ as a function of the Pr concentration *x*. (**d**) The conductivity component of the upturn as a function of temperature in the range of 1.7–40 K for *x* = 0.2. The solid line is the fitting curve using Eq. (5).

**Figure 5:**
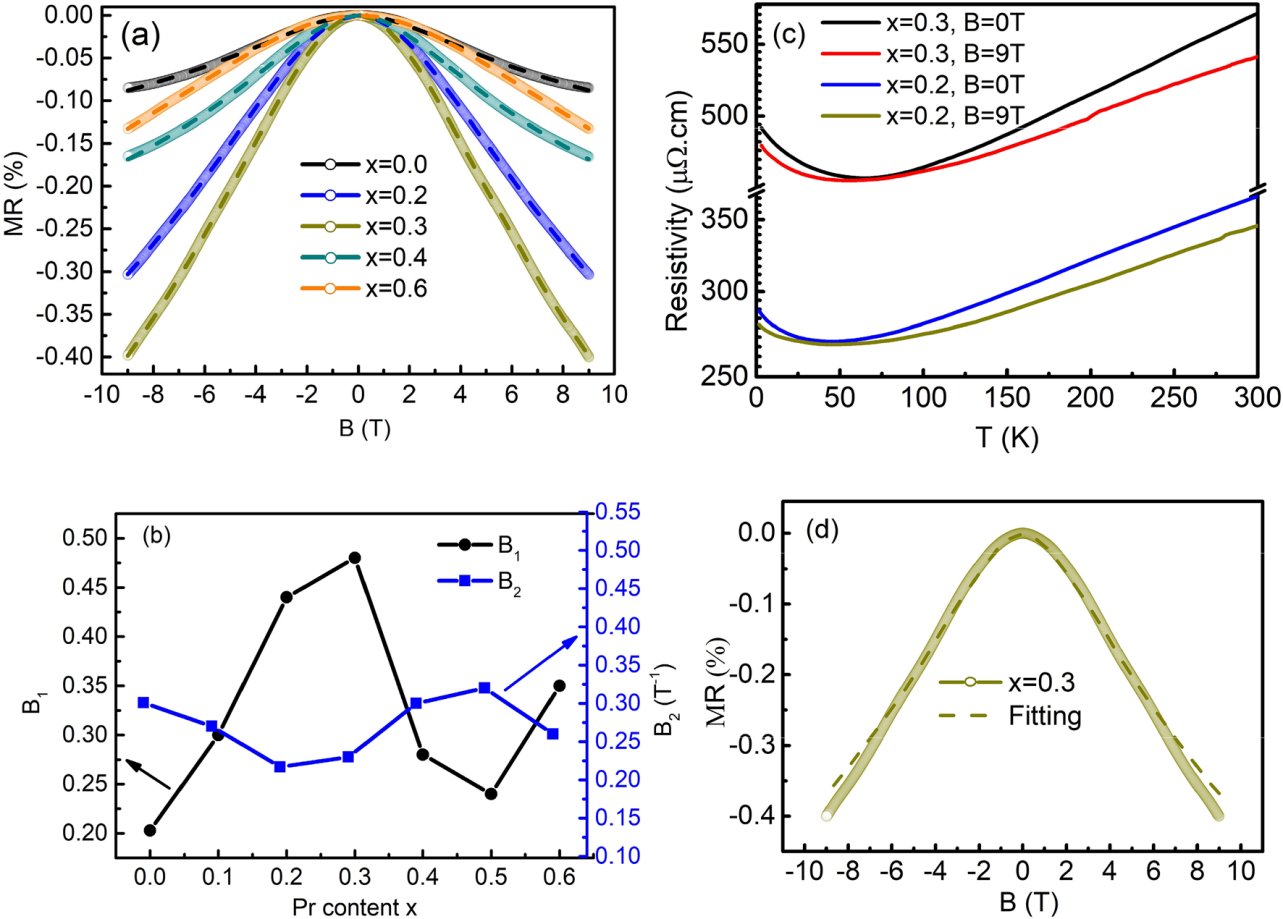
(**a**) *MR* at *T* = 1.7 K for the samples with *x* = 0.0, 0.2, 0.3, 0.4, and 0.6. The dashed lines are the curves fit using Eq. (9). (**b**) $B_{1}$ and $B_{2}$ parameters obtained by fitting experimental *MR* data to Eq. (9). (**c**) The resistivity as a function of temperature at *B* = 0 T and *B* = 9 T for *x* = 0.2 and *x* = 0.3, respectively. (**d**) *MR* at T = 1.7 K for *x* = 0.2, the dashed line is the curve fit using Eq. (6).

For *B* dependent resistivity,

6 $$\frac{\Delta \rho }{{\rho_{0} }} = \frac{{\rho_{H} - \rho_{0} }}{{\rho_{0} }} = \frac{{e^{2} \rho_{0} }}{{2\pi^{2} \hbar }}\left( {\frac{eB}{\hbar }} \right)^{1/2} \left[ {\left( {1 - \beta \left( T \right)} \right)f_{3} \left( {\frac{B}{{B_{\phi } }}} \right) - \frac{3}{2}f_{3} \left( {\frac{B}{{B_{2} }}} \right)} \right]$$

with $B_{\phi } = B_{in} + 2B_{S}$, $B_{2} = B_{in} + \frac{2}{3}B_{S} + \frac{4}{3}B_{SO}$, where the characteristic fields $B_{x} = \frac{\hbar }{{4eD\tau_{x} }}$, $\hbar$ is the reduced Plank’s constant, and subscript in, so, and s indicate the inelastic, spin–orbit, and magnetic spin-flip scattering times (fields), respectively. $\beta (T)$ is the term responsible for the Maki–Thompson correction. $f_{3} (1/x)$ is a function, which can be approximated as

$$f_{3} (1/x) = 2(\sqrt {2 + x} - \sqrt x ) - (0.5 + x)^{ - 1/2} - (1.5 + x)^{ - 1/2} + (2.03 + x)^{ - 3/2} /48$$

within 0.1% accuracy^41,42^. The magnitude of the spin–orbit field $B_{SO}$ is estimated to be small (0.01 T) and can be neglected. The fitting results using Eqs. (5) and (6) are displayed in Figs. 4d and 5d, respectively. Clearly, the fitting curves do not match the *T* and *B* dependence of resistivity data. This indicates that the WL is not the dominant effect for the low-T resistivity upturn in our samples.

Now we investigate the resistivity upturn based on other two remaining mechanisms, EEI and the Kondo effect. The Kondo effect, which was derived from a third-order perturbation theory, gives rise to a logarithmic variation in resistivity with *T*^1,43^, i.e. $- \rho_{K} \ln T$. The combination of Eqs. (1), (3) and the Kondo term leads to a total resistivity of

7 $$\rho = \rho_{SAT} (\rho_{0} + AT^{n} )/(\rho_{0} + AT^{n} + \rho_{SAT} ) - \rho_{k} \ln T,$$

where $\rho_{SAT}$, $\rho_{0}$, $A$, $n$ are parameters extracted from fitting in the metallic region. $\rho_{k}$ represents the contribution of the Kondo effect.

EEI plays a dominant role in strongly correlated electron systems and is enhanced by the strong disorder potential^38^. Altshuler et al.,^44^ pointed out that the EEI accompanying impurity scattering gives rise to a singularity in the density of states near the Fermi level, causing an anomalous *T* dependence of resistivity, i.e. $- T^{1/2}$ in the three-dimensional regime. Thus, the total resistivity including Eq. (1), Eq. (3), and the EEI term is given by

8 $$\rho = \rho_{SAT} (\rho_{0} + AT^{n} )/(\rho_{0} + AT^{n} + \rho_{SAT} ) - \rho_{e} T^{1/2} ,$$

where $\rho_{e}$ is the coefficient of the EEI term.

It seems that the data are well fit to both Eqs. (7) and (8). We plotted the experimental data of the sample with *x* = 0.2 in the temperature range from 1.7 K to 140 K with the fitting curve based on the Kondo effect (Fig. 4a). The EEI formulae yielded a similar fitting curve (not shown here), which was almost indistinguishable from the Kondo fitting curve.

For a more detailed comparison, we present the difference in curves between the experimental data and the theoretical curves based on the Kondo effect and the EEI effect (Fig. 4b) for the sample with *x* = 0.2. Additionally, the quality of the fitting is evaluated by comparing the parameter $\chi^{2}$ obtained for each equation. Here $\chi^{2}$ is defined as $\chi^{2} = \left( \frac{1}{N} \right)\sum\nolimits_{n}^{i = 1} {\frac{{(\rho_{raw}^{i} - \rho_{fit}^{i} )^{2} }}{{\rho_{fit}^{i} }}}$, where $\rho_{raw}^{i}$ and $\rho_{fit}^{i}$ are the experimental and theoretical resistivities at temperature $T_{i}$, respectively. The extracted $\chi^{2}$ values of the sample with *x* = 0.2 using Eqs. (7) and (8) were 0.9 × 10^–8^ and 4.7 × 10^–8^, respectively. The EEI case showed a relatively larger difference compared to the Kondo case, and this implies that the Kondo effect describes the experimental result better than the EEI effect. In addition, the *x* dependence of $\rho_{k}$ quite resembles that of the upturned part of the resistivity, which is defined as $\Delta \rho = (\rho_{{1.7\;{\text{K}}}} - \rho_{\min } )$, where $\rho_{1.7K}$ is the resistivity measured at 1.7 K and $\rho_{\min }$ is the minimum resistivity as shown in Fig. 4c. It can be clearly seen that $\Delta \rho$ is proportional to $\rho_{k}$ in good agreement with the previous report^45^.

Because the difference in the curve fittings is subtle, we carried out *MR* measurements from − 9 to 9 T at *T* = 1.7 K in order to additionally confirm the absence of the EEI effect in our samples. We define *MR* as $MR = \left( {\rho_{H} - \rho_{0} } \right){/}\rho_{0} \times 100$, where $\rho_{0}$ and $\rho_{H}$ are resistivities measured at *B* = 0 T and *H* T, respectively. Since *B* suppresses fluctuations in localized magnetic moments and spin-dependent scattering, a negative *MR* occurs in a Kondo system^46^. On the other hand, the EEI effect gives rise to a positive *MR* due to Zeeman splitting and orbital effects^47–50^.

In the present case, the *MR* data was found to be negative, supporting the Kondo effect as the main mechanism governing the low-*T* electrical phenomena in our samples. Here, Ni^2+^ ions, which did not participate in the regular lattice, were produced rather than Pr^3+^ ions, and the electrons released during oxygen vacancy formation became magnetic impurities. While the density of states from Pr^3+^ ions is far from the Fermi level, that from the Ni^2+^ ions is near it^51^. Thus, it is believed that Pr^3+^ merely increases structural disorder. Finally, to completely rule out the EEI effect in our samples, we performed the *T* dependence of resistivity measurement at *B* = 9 T with two samples of *x* = 0.2 and *x* = 0.3, which have large resistivity upturns as presented in Fig. 5c. The results show that the magnitude of upturns in the low-*T* resistivity decreases clearly under the applied *B*. This suppression is consistent with the Kondo theory. Therefore, from the analysis above, we fully confirm that the EEI effect is negligible in the low-*T* upturn of resistivity in our samples.

The *MR* at *T* = 1.7 K is plotted in Fig. 5a. The *MR* of all films was negative. Its absolute value increased and decreased with *x* at small and large *x*, respectively, with a maximum value around *x* = 0.3. This trend resembles the *x* dependence of the *c* lattice constant in Fig. 1b and that of the resistivity upturn in Fig. 3a.

To analyze the negative *MR*, we introduce the Khosla-Fisher model, which includes the effect of spin-dependent scattering. According to this model, the localized impurity spins are aligned in the direction of *B*. This effect lowers the scattering of conduction electrons by localized impurity spins, leading to a decrease in resistivity^52^. Several reports have successfully interpreted the negative *MR* using this model, in In_1−x_Mn_x_Sb films^53^, amorphous Cr-doped In_2_O_3_ films^54^, and (1−*x*)LNO + *x*CoFe_2_O_4_ (CFO) polycrystalline^55^. The semi-empirical formula of the negative *MR*^52^ given by Khosla and Fisher is expressed by

9 $$MR = \frac{{\rho_{H} - \rho_{0} }}{{\rho_{0} }} \times 100 = - B_{1}^{2} \ln (1 + B_{2}^{2} H^{2} )$$

This formula is based on the third-order expansion of the s-d exchange Hamiltonian which yields the logarithmic temperature variation of resistivity. This model explained the *T* dependence of the resistivity in dilute magnetic alloys^1,56^. The coefficients $B_{1}$ and $B_{2}$ are expressed by

10 $$B_{1} = A_{1} JN(\varepsilon_{F} )\left[ {S(S + 1) + \left\langle {M^{2} } \right\rangle } \right]$$

and

11 $$B_{2} = \left[ {1 + 4S^{2} \pi^{2} \left( {\frac{{2JN(\varepsilon_{F} )}}{g}} \right)^{4} } \right]\left( {\frac{{g\mu_{B} }}{{\alpha k_{B} T}}} \right)^{2} ,$$

respectively, where the parameter $A_{1}$ is the spin scattering contribution to total *MR*, $J$ is the exchange coupling constant, $N(\varepsilon_{F} )$ is the density of states at the Fermi level $\varepsilon_{F}$, $S$ and $g$ are the total spin and the effective Lande g factor of localized magnetic moment, respectively, $\left\langle {M^{2} } \right\rangle$ is the average of magnetization squared, and $\alpha$ is a numerical constant in the order of 1. $A_{1}$ is expressed as $AN_{A} (\sigma_{J} {/}\sigma_{0} )^{2}$, where $A$ is a coefficient, $N_{A}$ is Avogadro’s number, and $\sigma_{J}$ and $\sigma_{0}$ are the magnetic and nonmagnetic scattering cross sections, respectively.

This model was used to explain the *MR* of rare-earth metals^57^, in which the 4f electrons in the rare-earth ions are localized at the atomic sites. The *MR*s of our LPNO films are well fit to Eq. (9) as shown in Fig. 5a, where the dashed lines are the fitting curves. The *x* dependence of $B_{1}$ and $B_{2}$ are summarized in Fig. 5b. According to Eq. (11), $B_{1}$ is proportional to $A_{1} JN(\varepsilon_{F} )$. Since $\sigma_{J}$ and $\sigma_{0}$ are fixed in the given scattering mechanisms, $A_{1}$ will not vary much with *x*. Also, $N(\varepsilon_{F} )$ does not change with *x* even when a significant amount of oxygen vacancies exists. Therefore, the *x* dependence of $B_{1}$ reflects that of $J$.

According to Kondo theory, $\rho_{k} \sim zJ{/}\varepsilon_{F}$^1^, where *z* is the number of conduction electrons per atom. Thus, the relation of $B_{1} \sim \rho_{k}$ should hold. Indeed, we note that the *x* dependence of $B_{1}$ in Fig. 5b is quite similar to that of $\rho_{k}$ in Fig. 4c. This correlation supports $B_{1} \propto J$ in the present samples. Thus, the absolute values of the negative *MR* in the LPNO thin films are governed by $J$.

## Conclusion

In summary, the structural characteristics and the electric transport behaviors of La_1−x_Pr_x_NiO_3−δ_ (0 ≤ x ≤ 0.6) thin films were systemically studied. In particular, we found an intimate correlation between the *c* lattice parameter (or unit cell volume), and the magnitude of the resistivity upturn and magnetoresistance on the dependence of Pr concentration *x*. The *c* lattice parameter did not follow Vegard’s law because of the full in-plane strain, and the different amounts of oxygen vacancies in the samples; the *x* dependence of the *c* lattice parameter reflects the amount of oxygen vacancies. The resistivity upturn and magnetoresistance could be consistently explained by the Kondo effect. The analysis of the resistivity upturn and magnetoresistance demonstrate that their magnitudes are governed by the exchange coupling constant *J*.
